# Hiccups: Nerve Irritation or Masquerading as Acute Coronary Syndrome

**DOI:** 10.7759/cureus.48069

**Published:** 2023-10-31

**Authors:** Dessiree Cordero, Patrik Schmidt, Franklin Sosa, Maulin Patel, Eduard Sklyar

**Affiliations:** 1 Internal Medicine, BronxCare Hospital Center, Icahn School of Medicine at Mt. Sinai, New York, USA; 2 Internal Medicine, BronxCare Health System, New York, USA; 3 Cardiology, Mount Sinai Hospital/Bronxcare Health System, New York, USA; 4 Electrophysiology, BronxCare Health System, New York, USA

**Keywords:** cardiology, myocardial infarction, myocardial ischemia, acute coronary syndrome, hiccups

## Abstract

The typical clinical presentation of acute coronary syndrome (ACS) includes chest pain that may radiate to the left arm, shoulder, jaw, and neck, accompanied by diaphoresis, dyspnea, nausea, vomiting, and hiccups, which have been observed as the sole symptom of presentation. The mechanism of hiccups involves the activation of the vagus and phrenic nerves, leading to the activation of the diaphragm and intercostal muscles. Several hypotheses link hiccups to ACS, associating irritation of the left anterior descending artery with activation of sympathetic phrenic and vagal nerves. This case report highlights the occurrence of hiccups in patients with inferior and right ventricular myocardial infarction (MI), indicating possible nerve synapse involvement. Timely recognition of hiccups as a possible atypical symptom of ACS can facilitate early evaluation and management, preventing delays in patient care and ensuring better outcomes.

## Introduction

Acute coronary syndrome (ACS) continues to be one of the leading causes of death worldwide [1]. Many patients with ACS may present with classical symptoms such as substernal chest discomfort that worsens with physical or emotional stress and improves with rest or nitroglycerin; however, atypical presentations are also documented in the literature. While objective data to assess the presence of coronary atherosclerotic plaque formation and subsequent disruption, such as cardiac biomarkers, ischemic electrocardiographic changes, and regional wall motion abnormalities, is the frontline evaluation for most patients with suspicion of ACS, symptoms are considered more subjective information interpreted by providers differently. Hiccups, typically benign clinical symptoms, have been infrequently linked as a presenting symptom for acute coronary syndrome. We report a rare case of hiccups as a chief complaint that masquerades as ACS in an elderly male.

## Case presentation

We present a case of a 76-year-old male with a medical history significant for Mobitz type 2 heart block status (post-dual-chamber pacemaker placement), deep vein thrombus (DVT), pulmonary embolism (PE), osteoarthritis (OA), hyperlipidemia (HLD), chronic obstructive pulmonary disease (COPD), diet-controlled type 2 diabetes mellitus, and internal haemorrhoids, presenting to the emergency room with complaints of persistent, intractable hiccups for one day. The patient endorses that the day prior to the onset of his symptoms, he received a knee injection for chronic knee pain in the setting of his OA. Otherwise, he denies headaches, dizziness, shortness of breath, chest pain, abdominal pain, nausea, vomiting, diarrhoea, palpitations, chills, subjective fever, or night sweats.

His chest X-ray was negative for any acute intrathoracic pathology, and he was given a dose of metoclopramide and discharged from the ER. The following day, the patient began experiencing intermittent chest pain early in the morning hours, which awoke him from sleep. He described his chest pain as substernal, non-radiating, 9/10 in intensity, and pressure-like. He was given aspirin and nitroglycerin sublingually on the scene, with improvement in pain for approximately two hours. Besides his intermittent chest pain and hiccups, which have intermittently resolved but returned the previous night, the patient denied any other symptoms. Further questioning revealed no family history of any cardiovascular disease, personal history of tobacco or alcohol use, or drug use history.

**Figure 1:**
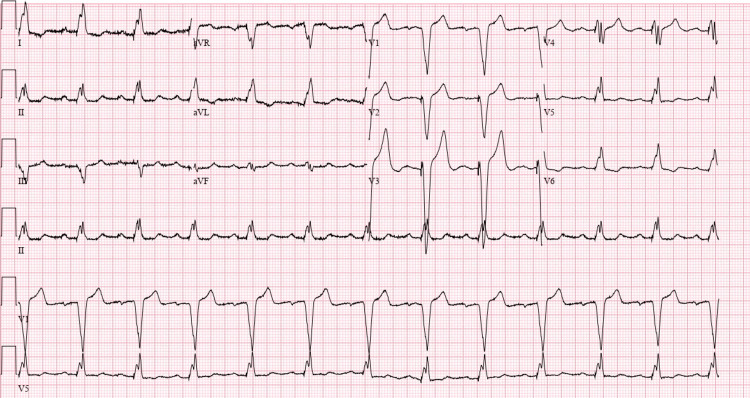
EKG on admission showing ventricular pacing with a rate of 73 beats per minute, PR interval of 210 ms, QRS duration of 160 ms, QTc of 484 ms, and normal axis.

On the initial exam, the patient was afebrile, saturating 97% oxygen on room air, with a respiratory rate of 17, a heart rate of 67, and a blood pressure of 137/87 mmHg. His initial laboratory findings revealed a reduced glomerular filtration rate of 52.1 ml/min/1.73 m^2^, lactic acid of 2.2 mmoles/L, and a troponin T level of 267 ng/L. Repeat labs showed an upward trend of lactic acid to 2.5 mmoles/L and a troponin T of 386 ng/L. His urine and toxicology reports were negative, except for mild proteinuria. The viral panel was negative for COVID-19 and other respiratory viruses. D-dimer was within normal limits. The chest X-ray from this visit showed no acute cardiopulmonary abnormality, while his ECG showed ventricular pacing with a rate of 73 beats per minute, a PR interval of 210 ms, a QRS duration of 160 ms, a QTc of 484 ms, and a normal axis (Figure 1). Due to the patient's presentation being suspicious for NSTEMI, the cardiac care unit (CCU) was consulted, and the patient was accepted. He received a loading dose of clopidogrel and was placed on a heparin drip. The TIMI score was 3.

After being admitted to the CCU, the patient was noted to have a hypertensive emergency with blood pressures ranging in the 190s/120s, which was promptly addressed using a nicardipine drip. Echocardiography showed grade 1 diastolic dysfunction with normal left ventricular ejection fraction and wall motion.

**Figure 2:**
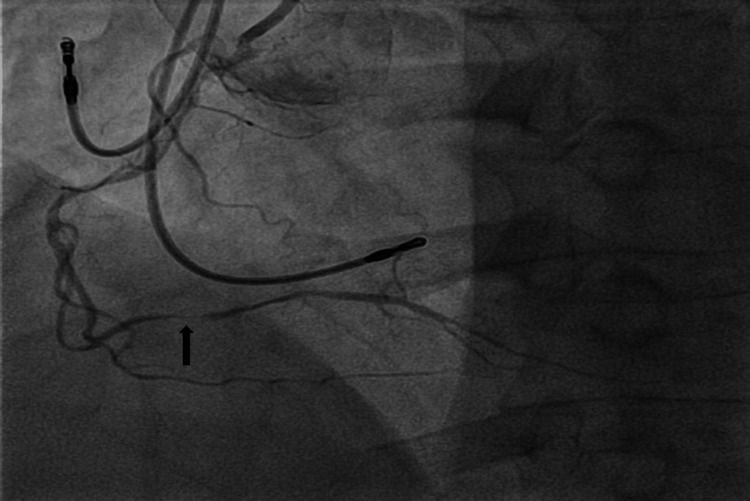
Coronary angiogram. Arrow points at the distal right coronary artery with 99% stenosis of 12 mm length.

**Figure 3:**
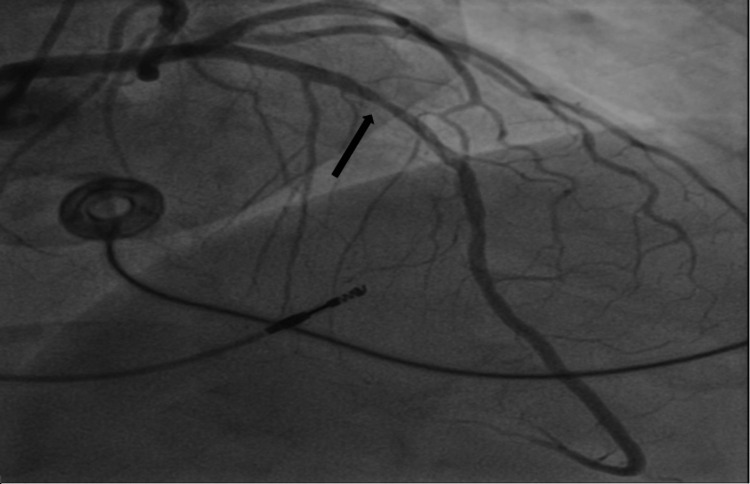
Coronary angiogram. Arrow points at middle left anterior descending artery with 12 mm 60% stenosis.

The patient underwent urgent left heart catheterization (LHC), which showed double vessel disease. The distal right coronary artery (dRCA) showed a 99% stenosis with a 12 mm length with TIMI 2 flow; this was felt to be the culprit lesion (Figure 2). Additionally, the middle left anterior descending artery (mLAD) showed 60% stenosis, 12 mm in length, with TIMI 3 flow (Figure 3). Plain old balloon angioplasty (POBA) was done on the dRCA with a 2.0 mm × 12 mm compliant balloon (small-vessel size). This led to a TIMI 3 flow post-procedure (Figure 5). No intervention was done on mLAD. Post-procedure, patients improved clinically, hiccups subsided, and they were eventually discharged home.

**Figure 4:**
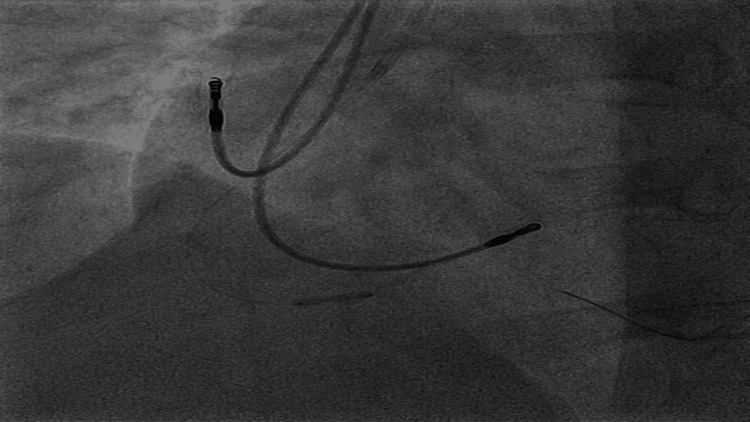
Coronary angiogram showing dRCA pre-POBA.

**Figure 5:**
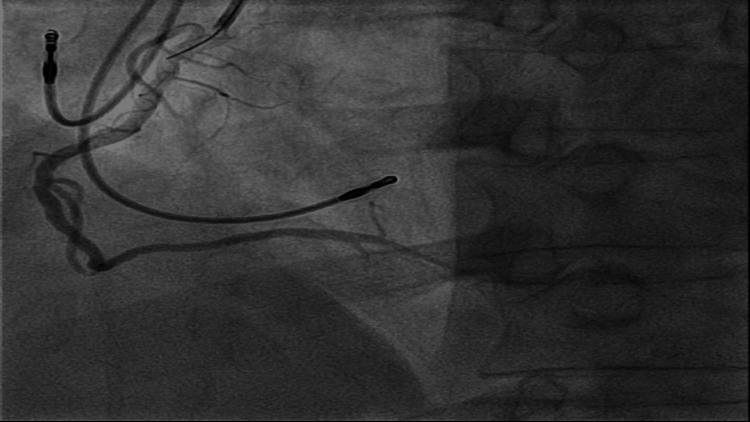
Coronary angiogram showing right coronary artery post-plain old balloon angioplasty with improvement of previous 99% stenosis.

Post-cardiac catheterization, the patient was advised to continue with aspirin 81 mg and clopidogrel 75 mg for a minimum of one year, with continuation of high-dose statin and close outpatient cardiology follow-up and monitoring.

## Discussion

ST-segment elevation myocardial infarction (STEMI) and non-ST-elevation myocardial infarction (NSTEMI), as well as unstable angina, typically include a myriad of symptoms such as substernal chest pain radiating to the left or right arm, shoulder, jaw, and neck, with associated diaphoresis, and/or dyspnea [2]. However, atypical symptoms like abdominal discomfort, nausea, emesis, syncope, seizures, and hiccups are also seen, especially in female patients [3].

The term hiccups is defined as a repetitive and involuntary muscle contraction of the diaphragm and intercostal muscle, followed by a closure of the larynx, which subsequently causes an unexpected blast of air hooked into the lungs, leading to the characteristic sound of “hic” [4]. It is usually transitory, but in certain conditions, it can last over 48 hours. When it is prolonged, as just mentioned, it is called persistent hiccups [5]. Hiccups are involuntary reflexes involving the diaphragm and central structures including the brainstem, temporal lobe, basal ganglia, hypothalamus, and spinal cord levels C3-5. They can occur due to various causes, including medullary strokes and pathologies affecting the central and peripheral nervous systems. While hiccups serve to protect the airways against esophageal aspiration, pathologic hiccups can be associated with conditions such as stroke, tumours, or myocardial infarction (MI) [6,7].

The actual mechanism of hiccups is not completely described or understood, but it is suspected that they consist of three parts. One afferent limb that includes vagus and phrenic nerves, a processor in the brain thought to be between the cervical spine and the brainstem, and one efferent limb carried by the phrenic nerve that activates the diaphragm and intercostal muscles [8].

Several hypotheses have been established about the mechanism of hiccups in ACS, including occlusion of the left anterior descending artery and activation of the afferent part of the sympathetic phrenic and vagal nerves [9,10]. Shaikh et al. [5] reported a case of a 74-year-old male who presented with persistent hiccups lasting for four days and was admitted for sepsis secondary to a diabetic foot ulcer. Coincidentally, during his evaluation, a routine 12-lead EKG revealed an inferior wall STEMI. Further investigation through diagnostic catheterization showed a complete RCA occlusion, confirming the cause of the myocardial infarction. A thallium viability study was performed, confirming nonviable myocardium, and consequently, he did not undergo percutaneous coronary intervention (PCI). In this case, the authors suggested that myocardial necrosis, which leads to debris and disintegrating particulate material, causes a persistent irritation of the vagus nerve, which consequently leads to persistent hiccups [5].

Various studies have shown that hiccups occur more frequently in patients with inferior MI and right ventricle infarction. This observation may be explained by the presence of interconnections between the phrenic nerve and the major part of the fibres reaching the pericardium from the right side [7,11]. Krysiak et al. [7] presented a case of a 62-year-old male with a history of recurrent hiccups and retrosternal pain upon physical exertion. On coronary angiography, the patient had left main coronary artery stenosis of 40% and significant stenosis on the LAD and posterolateral branch. Additionally, the RCA was significantly obstructed in 1/3 of the proximal-distal segment and critically obstructed in the end part. The patient underwent PCI with RCA angioplasty with subsequent clinical improvement, while hiccups associated with exertion were completely resolved [7]. Zhang et al. [12] reported a case of a 51-year-old African American with stent thrombosis post-balloon angioplasty with a stent of 99% stenosed mid-RCA. The patient’s primary symptom post-PCI was intractable hiccups for two days. Impressively, this patient’s troponins began rising after PCI at the exact same time as the hiccups began. The patient underwent balloon angioplasty with thrombectomy and new stent placement, with a resolution of hiccups.

Hovey et al. [13] presented a case of a 62-year-old male who presented to the emergency department with persistent hiccups for one week. Additionally, he reported progressively worsening shortness of breath and intermittent, burning epigastric pain for one week. An electrocardiogram revealed normal sinus rhythm with changes indicative of myocardial ischemia in lead aVL and significantly elevated troponin levels, leading to the diagnosis of NSTEMI. A coronary angiogram confirmed severe triple vessel disease, and the patient underwent coronary artery bypass graft (CABG) surgery. Following CABG, the patient's hiccups resolved, providing an interesting association between persistent hiccups and severe triple vessel disease [13]. This further shows that underlying coronary ischemia may have been the trigger for the hiccups, as reperfusion led to the resolution of symptoms.

While hiccups are not a common presentation of ACS, consideration of chest pain syndrome in these patients can lead to early identification and evaluation of ACS, which can ultimately avoid delays in patient care and protect patients from harm. In our case, hiccups were the only initial symptom, which, in the absence of other signs of ACS, was not the first differential diagnosis.

## Conclusions

Acute coronary syndromes continue to represent a common yet deadly clinical presentation worldwide. While typical symptoms such as chest pain, fatigue, and dyspnea are considered to be equivalents of an acute ischemic attack, atypical symptoms such as hiccups need to be considered as well in order to avoid underdiagnosing ACS, which can ultimately lead to increased patient morbidity and mortality. Including hiccups as a differential diagnosis of ACS will avoid a delay in diagnosis and possible early intervention.
